# Endosonographic monitoring of Sphinkeeper^®^ prostheses movements: does physical activity have an impact?

**DOI:** 10.1007/s13304-023-01636-y

**Published:** 2023-08-28

**Authors:** Christopher Dawoud, Daniel Gidl, Kerstin Melanie Widmann, David Pereyra, Felix Harpain, Berfin Kama, Stefan Riss

**Affiliations:** Division of Visceral Surgery, Department of General Surgery, Medical University Vienna, Waehringer Guertel 18-20, 1090 Vienna, Austria

**Keywords:** Faecal incontinence, Sphinkeeper^®^ prostheses dislocation, Prostheses migration

## Abstract

The Sphinkeeper^®^ procedure for treating faecal incontinence (FI) may be associated with potential implant migration (IM) and dislocation (ID), with considerable variations regarding their occurrence and effects on consecutive functional outcome. This study assessed IM and ID following the Sphinkeeper^®^ procedure and its correlation with physical activity. This was a prospective observational clinical study of ten patients undergoing Sphinkeeper^®^ operation due to FI between August 2020 and November 2020 at the Medical University of Vienna. Patients were followed-up after 1, 2, 3 and 6 months and 1 year postoperatively. Each follow-up visit included endosonographic monitoring of protheses location and manometric examinations. Additionally, functional outcome and physical activity were assessed using validated standardized questionnaires. The median number of prostheses implanted was 10 (IQR 9–10). The St. Mark’s incontinence (SMS) score improved significantly until the last follow-up (p = 0.049), without observing a significant effect on the physical SF-12 score. The median rate of implants leading to IM and ID was 3 (range 1–4) and 2 (range 1–2) after 3 months of follow-up. A strong association of deltaSMS with number of dislocated prostheses at one month after Sphinkeeper^®^ implantation was observed (r = 0.654, p = 0.078). Physical activity, assessed by the international physical activity questionnaire, did not have an impact on the correct prosthesis placement (1 month: p = 0.527; 2 months: p = 0.886; 3 months: p = 0.180; 6 months: p = 0.111). IM and ID of Sphinkeeper^®^ prostheses occurred frequently early after surgery and affected functional outcome negatively. Physical activity did not have an influence on the implants displacement.

## Introduction

Fecal incontinence (FI) describes the involuntary loss of solid and liquid fecal matter or intestinal gas. It is categorized into active and passive FI, defined by perceived and unperceived loss of stool, respectively [1]. The overall prevalence of FI has been shown to reach up to 7.7% of the general population, without comprising leakage of gas [1–3]. The influence of FI on quality of life is substantial, but still many individuals experiencing FI do not seek medical attention for treatment [4]. Etiologies of FI are diverse and include structural damage of the sphincter apparatus, neural damage or functional disorders [4–6].

If conservative treatment fails, surgery can be a valuable option. Although several surgical techniques exist, the success rates vary significantly and some patients do not respond at all [7–12].

One minimal invasive approach represents the injection of so-called bulking agents, resulting in a potential increase of intra-anal resting pressure and a compensation of anal asymmetries. This technique has shown promising initial functional results with disappointing long-term success, likely due to displacement or resorption of the semi-soluble substances used [13–15].

Based on this concept, the Gatekeeper^®^ procedure was introduced, which was further modified to the Sphinkeeper^®^ procedure, utilizing 10 larger instead of 6 smaller cylindrical semi-solid implants. Within 48 h of insertion into the intersphincteric groove, the implants expand their original volume by up to 720% as a result of water absorption [16, 17]. This approach has been shown to be a safe method with long-term efficacy in up to 50% of patients [18, 19]. Notably, implant migration (IM) and dislocation (ID) are common after this procedure, with controversial data on its onset and impact on functional outcome [16–18, 20–27].

The current study aimed to evaluate the timing of IM and ID and associated risk factors following Sphinkeeper^®^ implantation.

## Methods

At a single tertiary referral center, ten patients with FI were prospectively recruited and underwent the Sphinkeeper^®^ surgery. The Medical University of Vienna’s regional Ethics Committee granted its approval.

Patients who were older than 18 and reported FI (incontinence for liquid and/or solid stool) for at least six months were comprised. All patients were treated conservatively, which included diet modifications, medication to regulate stools, pelvic floor exercises, and biofeedback therapy for three months. Exclusion criteria were anal fistulas, local malignant diseases and inflammatory bowel disease.

Patients were followed-up after 1, 2, 3 and 6 months and 1 year postoperatively.

### Outcome measures

All patients received an endoanal ultrasound examination (EAUS; Flex Focus 500, BK Medical Holding Company, Inc.) to investigate internal anal sphincter (IAS) and external anal sphincter (EAS) configuration. IM and ID were recorded at each visit. The position was considered correct if more than 50% of the prostheses showed correct placement (no IM or ID) at the same vertical level. The movement inside the intersphincteric space was categorized as dislocation, and movement through the muscle or out of the intersphincteric space as migration.

To analyse resting, squeezing, and straining pressure anorectal manometry (ARM) (THD^®^ Anopress, Pressprobe, Sensyprobe) was performed in each participant. Compliance and sensitivity of the anorectum were assessed by balloon testing.

Functional outcome was evaluated using standardised validated questionnaires. The St. Mark’s incontinence score was used to assess FI and the Short-Form-Health Survey (SF-12) was used to evaluate the quality of life [28, 29]. The international physical activity questionnaire (IPAQ) was conducted in order to evaluate the effect of activity on the occurrence of ID and IM.

### Surgical technique

According to the prior description, the surgical implantation was carried out in the lithotomy position [27]. All procedures were performed or supervised by a colorectal consultant. Patients received antibiotic single-shot prophylaxis (Cefuroxime 1.5 g and Metronidazole 1.5 g) and a preoperative enema (Klistier^®^ Fressenius, 130 ml). In order to ensure 24 h of postoperative bed rest, a urinary catheter was implanted. Two cm lateral to the anus, 9–10 two mm skin incisions were created. The delivery system was then used to place the prostheses in the intersphincteric space. The endoanal sonography was performed in all patients to determine the exact location. All prosthesis underwent the identical process across the entire circumference. Absorbable sutures were used to close the skin incisions.

### Statistical analysis

Statistical analysis was performed using the SPSS statistical software package (IBM SPSS Statistics for Mac, Version 26.0). For descriptive statistics the Shapiro–Wilk test was applied to evaluate normal distribution of the data. Whenever a normal distribution was identified, the mean and standard deviation were reported. Otherwise, the interquartile range and the median were mentioned. Accordingly, Students t-test or Friedman signed rank tests were applied as appropriate. Consequently, non-parametric tests were used for the exploratory statistics due to the sample size of n = 10. Here, continuous variables are expressed either as median with interquartile range (IQR) or mean with standard deviation (SD), as suitable. Categorical variables are presented as numbers with percentages. Quantitative variables were compared using Mann–Whitney U test or Wilcoxon test. To explore dichotomous variables, Chi-squared test was used. A p value < 0.05 was considered to denote statistical significance.

### Study registration

This study was registered at ClinicalTrials.gov (NCT04992429).

## Results

### Patient characteristics

Between August 2020 and November 2020, ten patients (9 women and 1 men) suffering from FI were treated using the Sphinkeeper^®^. The median follow-up time was 22.0 months (IQR 12.0–26.0). Table 1 provides an overview of the patient demographics. The median duration of FI until surgery was 98 months (IQR 64.3–173.8).

**Table 1:** Demographics and baseline characteristics

	n = 10
Demographics	
Age [years], median (IQR)	74.0 (67.5–79.3)
Female sex, n (%)	9 (90.0)
BMI [kg/m^2^], median (IQR)	25.8 (25.0–29.4)
Clinical history	
History of smoking n (%)	3 (30.0)
Childbirth, n (%)	7 (70.0)
Perineal tear, grade II, n (%)	5 (50.0)
Previous pelvic floor surgery, n (%)	8 (80.0)
Hysterectomy, n (%)	5 (50.0)
Sphincteroplasty surgery, n (%)	1 (10.0)
Rectopexy, n (%)	1 (10.0)
Internal sphincter defect, n (%)	2 (20.0)
External sphincter defect, n (%)	3 (30.0)
Combined sphincter defect, n (%)	4 (40.0)
FI form	
Active FI, n (%)	6 (60.0)
Passive FI, n (%)	1 (10.0)
Mixed FI, n (%)	3 (30.0)
FI episodes	
Daily, n (%)	6 (60.0)
3×/week, n (%)	2 (20.0)
1–2×/week, n (%)	2 (20.0)
FI cause	
Idiopathic, n (%)	4 (40.0)
Iatrogen after surgery or radiation, n (%)	3 (30.0)
Gynecological trauma, n (%)	2 (20.0)
Neurological disease, n (%)	1 (10.0)

The demographic and baseline characteristics of included patients;

*BMI* body mass index

### Surgical outcome

The median number of prostheses implanted was ten (IQR 9–10). The median operative time for ultrasound-guided implantation of the prostheses was 35.0 min (IQR 33.8 – 53.0), without intraoperative complications.

Patients spent a median of 2 days in hospital, with an interquartile range of 2–3 days. The postoperative course was uneventful in all patients.

### Functional outcome

**Fig. 1:**
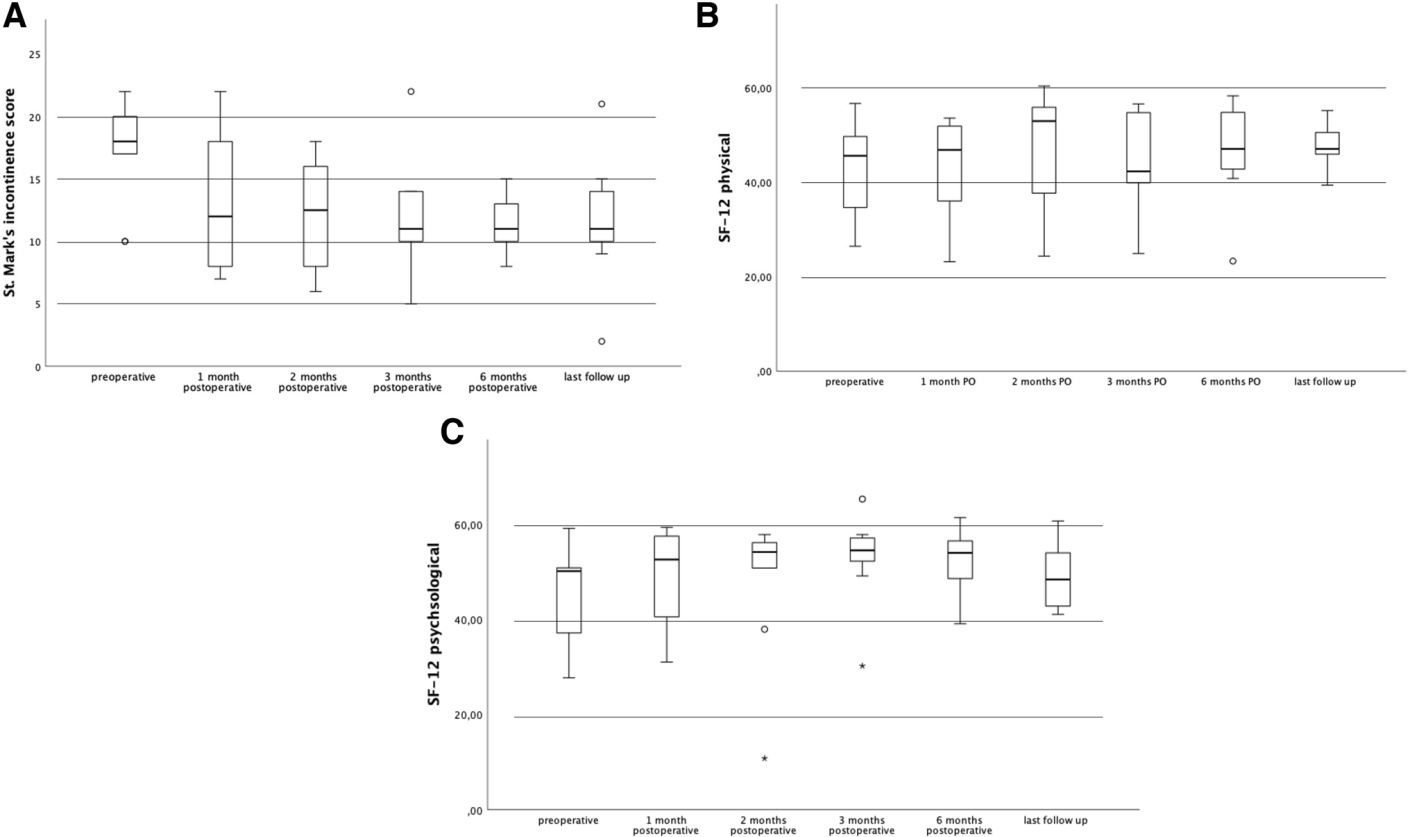
Outcome measurements. The figure shows the course of parameters from preoperative to 1, 2, 3, 6 months, and the last follow-up visit. **A** St. Mark’s incontinence score, Physical SF-12, **C** Mental SF-12. *p < 0.05

The St. Mark’s incontinence score decreased significantly after surgery (median preoperative = 18 (IQR 17–22); median one month PO = 12 (IQR 8–19), p = 0.035), and was significantly lower until time of last follow (compared to preoperative: median 2 months PO = 13 (IQR 8–16), p = 0.042; median 3 months PO = 11 (IQR 10–14), p = 0.036; median 6 months PO = 11 (IQR 10–14), p = 0.012; median last follow-up = 11 (IQR 10–15), p = 0.049; Fig. 1A), indicating that FI significantly improved following Sphinkeeper^®^ implantation.

The physical SF-12 score was not significantly affected by Sphinkeeper^®^ implantation (median preoperative = 45.6 (IQR 31.3–51.1); median 1 month PO = 46.9 (IQR 34.3–52.1); median 2 months PO = 53.0 (IQR 34.5–56.7); median 3 months PO = 42.3 (IQR 36.4–55.1); median 6 months PO = 47.1 (IQR 41.8–55.6); median last follow-up = 47.1 (IQR 42.7–52.9) p = 0.393; Fig. 1B).

In contrast, the psychological SF-12 as an indicator of quality of life showed a transient improvement throughout the follow-up (median preoperative = 50.1 (IQR 36.9–53.4); median 1 month PO = 52.6 (IQR 39.0–57.5), p = 0.110; median 2 months PO = 54.2 (IQR 47.6–56.6), p = 0.028; median 3 months PO = 54.5 (IQR 50.7–57.5), p = 0.025; median 6 months PO = 54.0 (IQR 45.8–58.1), p = 0.208; median last follow-up = 48.4 (IQR 41.9–57.3), p = 0.715; Fig. 1C).

During patient follow-up, no significant dynamics with regard to anal manometric tests and balloon tests were observed (Table 2).

**Table 2:** Anorectal manometry and sensitivity evaluation

n = 10	Preoperative	Follow-up 1 month	Follow-up 2 months	Follow-up 3 months	Follow-up 6 months	Last follow-up	p*
Anorectal manometry							
Resting pressure [mmHg], median (IQR)	22.5 (10.8–26.3)	23.5 (16.8–27.0)	21.0 (17.5–32.0)	24.0 (15.8–34.0)	27.0 (17.5–30.0)	23.0 (14.5–30.0)	0.258
Squeezing pressure [mmHg], median (IQR)	31.0 (26.8–42.5)	38.0 (34.0–59.3)	38.0 (30.5–52.0)	38.0 (30.3–47.3)	40.0 (32.0–51.0)	34.0 (23.5–53.0)	0.036*
Straining pressure [mmHg], median (IQR)	26.0 (19.8–46.5)	29.5 (26.5–49.3)	36.0 (27.5–53.5)	32.0 (20.0–39.0)	38.0 (21.0–45.5)	23.0 (16.5–32.0)	0.228
Anorectal sensitivity							
First anorectal sensation [ml], median (IQR)	69.0 (30–117.5)	37.5 (18.0–86.3)	40.0 (22.5–55.0)	27.5 (20.0–41.3)	20.0 (15.0–45.0)	30.0 (17.5–170.0)	0.319
Urge to defecate [ml], median (IQR)	90.0 (50.0–132.5)	82.5 (41.3–112.5)	85.0 (52.5–95.0)	60.0 (47.5–81.3)	60.0 (50.0–70.0)	60.0 (32.5–192.5)	0.533
Maximal tolerated volume [ml], median (IQR)	122.5 (80.0–180.0)	113.5 (71.3–170.0)	110.0 (85.0–120.0)	95.0 (71.3–115.0)	90.0 (80.0–100.0)	100.0 (47.5–225.0)	0.398

The results of anorectal manometry (mmHg) and sensitivity (ml) before and after surgery using Anopress^®^ and Sensyprobe^®^

*p = Wilcoxon test for connected samples between the mean of the preoperative results and of the last follow-up examination

#### Relevance of surgical success for patient outcome

We further evaluated which surgical parameters were associated with subjective improvement, defined as the delta of the St. Marks score from baseline until the last follow-up (deltaSMS).

The number of implanted prostheses did not significantly correlate with deltaSMS (r = -0.383, p = 0.349). A strong association of deltaSMS with number of dislocated prostheses at one month after Sphinkeeper^®^ implantation was observed (r = 0.654, p = 0.078).

#### Movements of prostheses and the impact of physical activity

Patients’ details including the number of migrated and dislocated prostheses as well as the number of correct prostheses at the same level are outlined in Table 3.

**Table 3:** Endosonographic evaluation of prostheses placement

n = 10	Follow-up 1 month	Follow-up 2 months*	Follow-up 3 months**	Follow-up 6 months*	Last follow-up
Prostheses evaluation					
Patients with correctly placed prostheses, n (%)	7 (70.0)	5 (55.6)	5 (71.4)	6 (66.7)	6 (60.0)
Number same height = correctly placed, median (IQR)	7 (5–7.25)	6 (5–7)	6 (5–7)	6 (5–7)	6 (5–7)
Patients with dislocated prostheses, n (%)	7 (70.0)	8 (88.9)	6 (85.7)	6 (66.7)	6 (60.0)
Number of dislocated prostheses, median (IQR)	1.5 (0–2.25)	2 (1–2.5)	2 (1–2)	1 (0–1)	1 (0–1)
Patients with migrated prostheses, n (%)	6 (60.0)	7 (77.8)	7 (100.0)	8 (88.9)	8 (80.0)
Number of migrated prostheses, median (IQR)	1.5 (0–3)	3 (1–3)	3 (1–4)	1 (1–3.5)	1 (1–3.5)

This table shows the prostheses location at 1, 2, 3, 6 months and at the last follow-up after Sphinkeeer^®^ implantation. Correctly placed prostheses were defined as more than 6 prostheses at the same vertical level, at the level of the upper and middle thirds of the anal canal and inside the intersphincteric space. The movement of each prothesis along the intersphincteric space was defined as dislocation, the movement through the sphincteric muscle or out of the intersphincteric space as migration. *Missing data: n = 9; ** missing data: n = 7

In addition, patients were grouped into 4 categories according to the correct or incorrect position of the prostheses at the time of outpatient visits.

**Fig. 2:**
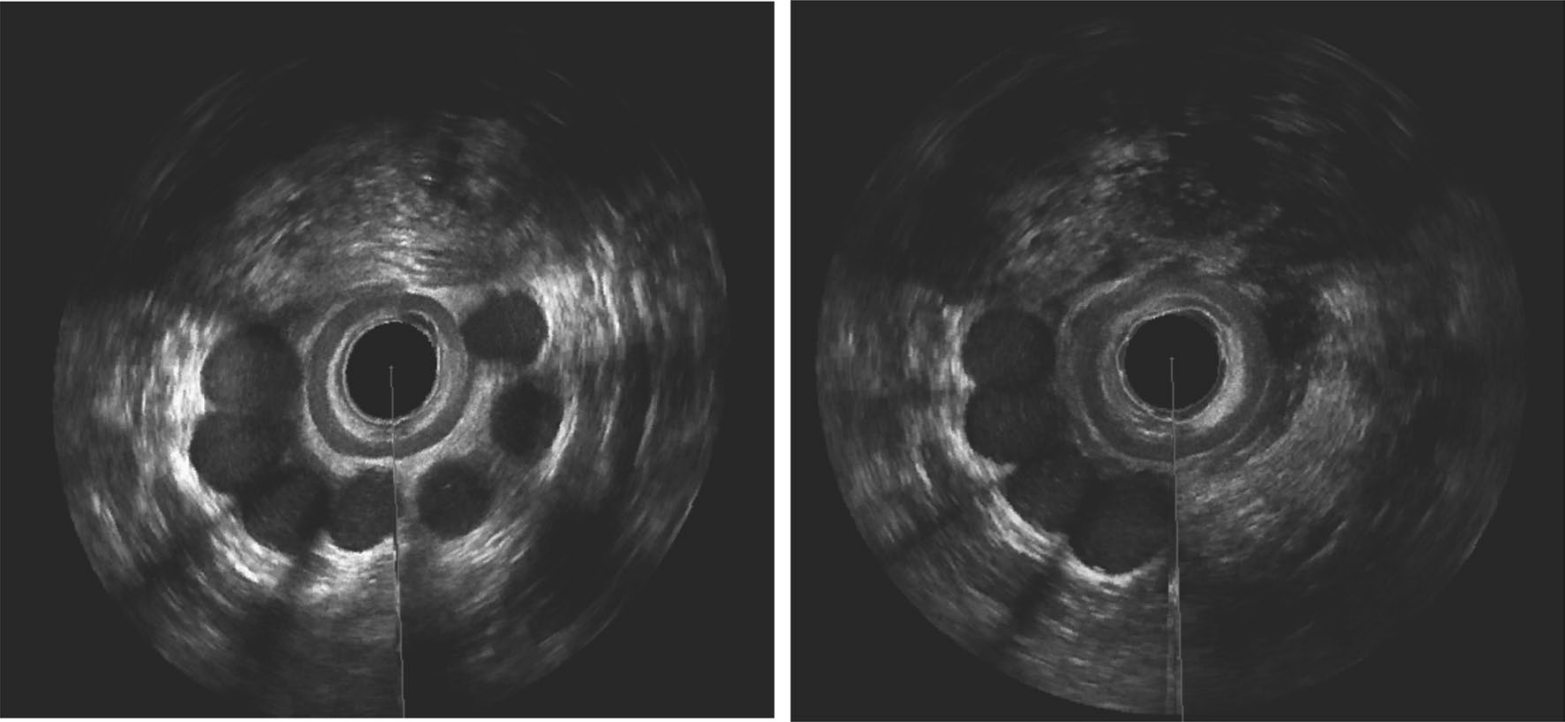
**A** 3D anal Endosonography 1 month postoperatively: 7 prostheses in the intersphincteric space in correct postion. **B** 3D anal Endosonography 12 months postoperatively: 4 prostheses in the intersphincteric space in correct position, the other 3 prostheses are not seen in same place as before

**Fig. 3:**
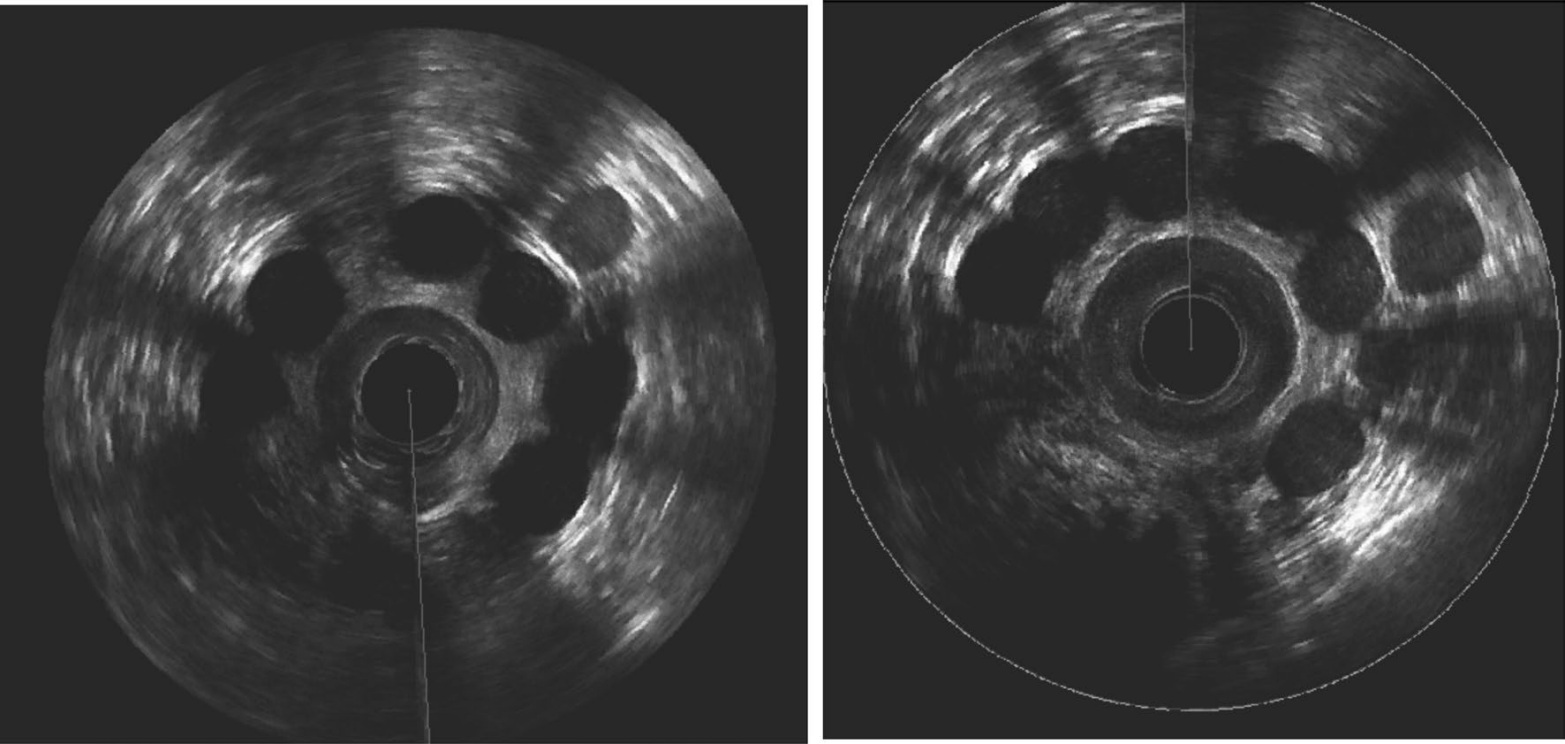
**A** 3D anal Endosonography 1 month postoperatively: 6 prostheses in the intersphincteric space in correct postion, 1 prostheses migrated outside of the intersphincteric space. **B** 3D anal Endosonography 16 months postoperatively: 6 prostheses in the intersphincteric space in correct postion, the distace between the prostheses at 10–12’o clock shortened, 1 prostheses migrating inside the intersphincteric space, 1 prostheses sill outside the intersphincteric space

Patients with a majority of good localized prostheses at baseline and at the end of follow-up were classified as category I (n = 5) (Figs. 2A, B and 3A, B).

The majority of these patients showed a good response in the form of a low deltaSMS score, indicative of a good improvement in the St. Mark’s incontinence score.

Patients in whom the majority of the localized prostheses was incorrect at the beginning of the examination and in whom a good localization was achieved during the further controls were assigned to category II (n = 2). Patients in this group showed the best symptoms improvement in terms of change in deltaSMS.

Category III defined patients initially with correct prostheses location and with worse location at the last FU (n = 1).

**Fig. 4.**
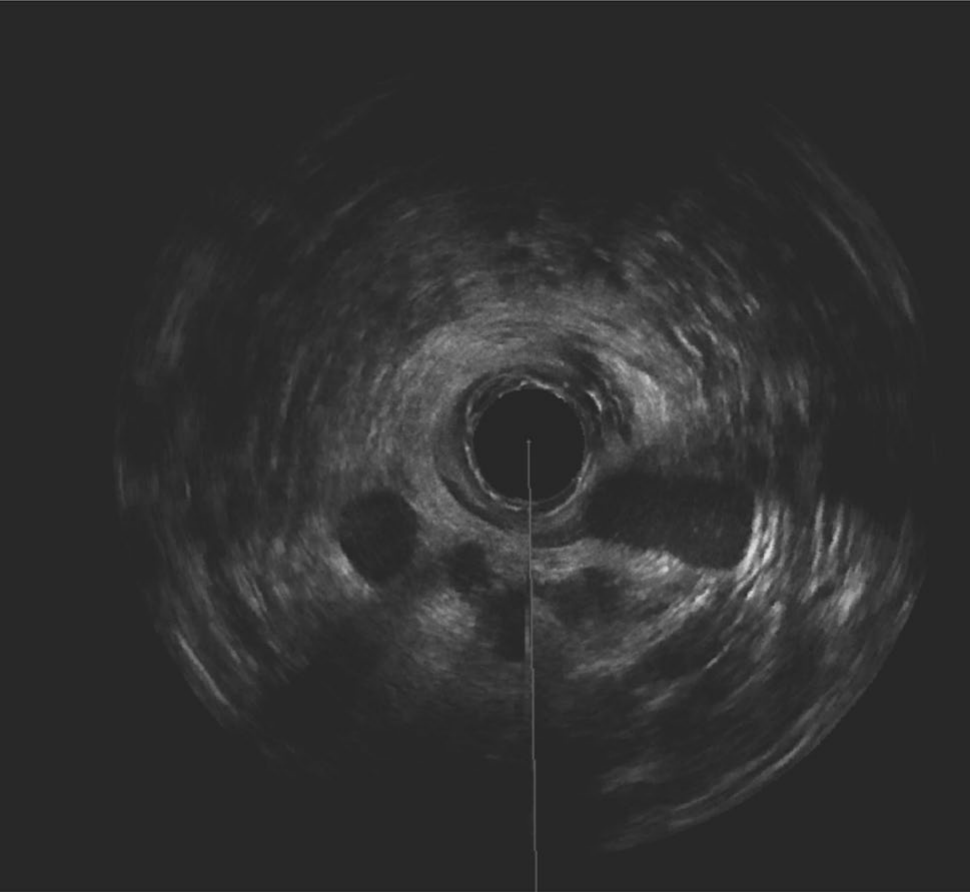
3D anal endosonography 1 month postoperatively: dislocation of 1 prosthesis at 8’o clock, in a 90° to the anal canal

Category IV included prostheses that performed poorly throughout the short-term and long-term follow-up (n = 2) (Fig. 4).

Physical activity, evaluated by the IPAQ, did not have an impact on the correct prosthesis placement (1 month: p = 0.527; 2 months: p = 0.886;3 months: p = 0.180;6 months: p = 0.111). There was also no statistical significance in relation to migrated or dislocated prostheses to the IPAQ. (ID: 1 month: p = 0.081; 2 months: p = 0.390; 3 months: p = 0.448; 6 months: p = 0.385; IM: 1 month: p = 0.303; 2 months: p = 0.241; 3 months: p = 0.104; 6 months: p = 0.188).

**Fig. 5:**
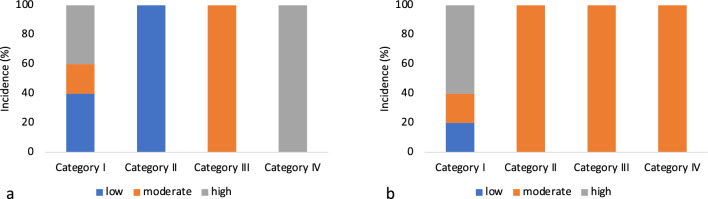
Categorization prostheses movement and IPAQ

However, we found that correct position (Category I and II) led to an increase in physical activity, which seemed most likely to be explained by better continence. In Category I, 20% of the patients improved their physical activity and reached a moderate activity level. Another 20% managed an activity improvement to the highest level. In contrast, patients in categories III and IV showed a decreasing trend in physical activity in the follow-up. Here, a downgrading of physical activity to the worst level occurred in both patients in this group (Fig. 5A, B).

## Discussion

In the present study, we aimed to assess the occurrence and timing of migration of the Sphinkeeper^®^ prostheses and its effect on the postoperative function. In addition, we evaluated whether physical activity, defined by a standardized scoring system, does have an effect on the dislocation rate.

Notably, we found that ID and IM are common problems and do occur early after surgery. Surprisingly, the amount of physical activity postoperatively did not have a significant effect on the Sphinkeeper^®^ prostheses movement rate. This is an important finding, which needs to be discussed with the patient.

Although migration has been reported by several studies, complications were rare events after surgery [26, 30]. Most studies did not report infections or perforations; thus, the procedure can be regarded a particularly safe. Only one study observed an abscess, which required surgical drainage [31]. One patient was treated by a removal of 2 prostheses, due to dislocation near the skin site, causing minor discomfort to the patient. Surgical removal was conducted without difficulties [27].

Patients undergoing Sphinkeeper^®^ surgery showed significant improvement in FI symptoms in several trials [21, 25–27, 32]. Furthermore, data suggested that the procedure resulted in long lasting reduction of FI compared to other bulking agents, although direct comparisons are missing.

Since the Sphinkeeper^®^ procedure is a novel method, long-term studies are still missing, but are mandatory to approve this assumption.

A number of studies have reported on IM or ID during their follow-up. Noteworthy, the definition of implant movements varies and a clear description and differentiation between IM and ID are hardly available. In addition, available data are conflicting whether IM and ID were associated with worse functional outcome.

In the first study on the Gatekeeper^®^ procedure, Ratto et al. did not find evidence of IM among 14 included patients within 90 days after the procedure [17]. In a subsequent trial, they observed dislodgement of a single prosthesis in only 3 out of 54 patients, although criteria for correct implant location during the follow-up were not clearly defined [20]. In the initial study on the Sphinkeeper^®^ procedure involving 10 patients, Ratto et al. identified a distal dislocation of a single prosthesis within the intersphincteric space one week after the procedure. However, no further instances of IM or ID were observed on endoanal ultrasound within 3 months of follow-up [16]. In contrast, de la Portilla et al. reported on IM/ID in a total of 24 out of 42 prostheses implanted in 5 out of 7 patients following the Gatekeeper^®^ procedure. Nevertheless, there was no clear effect of IM/ID on therapeutic success [22].

Leo et al. found only seven out of ten prostheses visible on imaging after a follow-up of 6–12 months, with a median of only 5 prostheses ideally placed. They observed no association between the number of correct placed prostheses and a favorable functional outcome [25]. Litta et al. reported the presence of at least six out of ten prostheses within the target area in 23 out of 42 patients after the Sphinkeeper^®^ procedure. Adequate prosthesis location at the last follow-up was significantly associated with better success [21]. La Torre et al. observed the extrusion of one prosthesis in two patients and displacement of one prosthesis in one patient. However, they did not observe a connection between this dislodgement and functional outcome [26].

A re-do procedure involving the replacement of lost implants has been successfully attempted. However, there is insufficient data to generally support this approach in terms of complications, efficacy, and the risk of repeated IM or ID [17, 18, 20, 33].

The results of our current study demonstrated a median rate of IM and ID of two and three implants. IM and ID predominantly occurred within the first months following placement, with minimal changes in implant location thereafter. Furthermore, the results indicated a possible correlation between the number of correctly placed implants and the reduction of FI. Furthermore, based on our data, we cannot recommend reducing physical activity postoperatively as a measure to minimize the likelihood of IM or ID.

Few limitations should be addressed in this study, which pertain to the short median follow-up period of 22 months, the limited number of patients included, the single-center nature of the study, and the focus on functional disease rather than concrete objective measures. It is crucial to acknowledge the brevity of the follow-up period, as it may not provide a definitive answer regarding the occurrence of delayed IM or ID following the Sphinkeeper^®^ procedure. While the present study adopted an observative approach, this design choice, coupled with the small sample size, inevitably reduces the statistical power and generalizability of the findings. Additionally, it is important to note that this study primarily focused on functional disorders rather than providing concrete hard facts regarding the efficacy and long-term outcomes of the Sphinkeeper^®^ procedure. To address these limitations comprehensively, it is necessary to expand the research scope by incorporating larger patient cohorts, multi-center collaborations, extended follow-up periods, and a comprehensive evaluation of objective clinical parameters to ascertain the true impact of the Sphinkeeper^®^ procedure on the development of delayed IM or ID.

## Conclusion

The Sphinkeeper^®^ represents a promising therapeutic option in the management of fecal incontinence (FI), demonstrating significant functional improvement in our cohort. While the number of implanted prostheses did not significantly correlate with the patients outcome, correct prosthesis placement was associated with better symptom improvement. Notabyl, the occurrence of IM and ID was not increased by higher physical activity.

## Data Availability

The raw datasets generated during and/or analyzed during the current study are not publicly available due to the sensitive nature of the questions asked in this study but are available from the corresponding author at reasonable request.

## References

[1] Desprez C, Turmel N, Chesnel C, Mistry P, Tamiatto M, Haddad R (2021). Comparison of clinical and paraclinical characteristics of patients with urge, mixed, and passive fecal incontinence: a systematic literature review. Int J Colorectal Dis.

[2] Ditah I, Devaki P, Luma HN, Ditah C, Njei B, Jaiyeoba C (2014). Prevalence, trends, and risk factors for fecal incontinence in united states adults, 2005–2010. Clin Gastroenterol Hepatol.

[3] Ng KS, Sivakumaran Y, Nassar N, Gladman MA (2015). Fecal incontinence: Community prevalence and associated factors—a systematic review. Diseases of the colon and rectum.

[4] Aitola P, Lehto K, Fonsell R, Huhtala H (2010). Prevalence of faecal incontinence in adults aged 30 years or more in general population. Colorectal Dis.

[5] Lazarescu A, Turnbull GK, Vanner S (2009). Investigating and treating fecal incontinence: when and how. Can J Gastroenterol.

[6] Rey E, Choung RS, Schleck CD, Zinsmeister AR, Locke GR, Talley NJ (2010). Onset and risk factors for fecal incontinence in a US community. Am J Gastroenterol.

[7] Bharucha AE, Rao SSC, Shin AS (2017). Surgical interventions and the use of device-aided therapy for the treatment of fecal incontinence and defecatory disorders. Clin Gastroenterol Hepatol.

[8] Boyle DJ, Knowles CH, Lunniss PJ, Scott SM, Williams NS, Gill KA (2009). Efficacy of sacral nerve stimulation for fecal incontinence in patients with anal sphincter defects. Dis Colon Rectum.

[9] Faucheron JL, Voirin D, Badie B (2010). Sacral nerve stimulation for fecal incontinence: causes of surgical revision from a series of 87 consecutive patients operated on in a single institution. Dis Colon Rectum.

[10] Thaha MA, Abukar AA, Thin NN, Ramsanahie A, Knowles CH (2015). Sacral nerve stimulation for faecal incontinence and constipation in adults.

[11] Carrington EV, Evers J, Grossi U, Dinning PG, Scott SM, O’Connell PR (2014). A systematic review of sacral nerve stimulation mechanisms in the treatment of fecal incontinence and constipation.

[12] Madoff RD, Rosen HR, Baeten CG, Lafontaine LJ, Cavina E, Devesa M (1999). Safety and efficacy of dynamic muscle plasty for anal incontinence: lessons from a prospective, multicenter trial. Gastroenterology.

[13] Graf W, Mellgren A, Matzel KE, Hull T, Johansson C, Bernstein M (2011). Efficacy of dextranomer in stabilised hyaluronic acid for treatment of faecal incontinence: a randomised, sham-controlled trial. Lancet.

[14] Watson NFS, Koshy A, Sagar PM (2012). Anal bulking agents for faecal incontinence. Colorectal Dis.

[15] Guerra F, La Torre M, Giuliani G, Coletta D, Amore Bonapasta S, Velluti F (2015). Long-term evaluation of bulking agents for the treatment of fecal incontinence: clinical outcomes and ultrasound evidence. Tech Coloproctol.

[16] Ratto C, Donisi L, Litta F, Campennì P, Parello A (2016). Implantation of SphinKeeperTM: a new artificial anal sphincter. Tech Coloproctol.

[17] Ratto C, Parello A, Donisi L, Litta F, De Simone V, Spazzafumo L (2011). Novel bulking agent for faecal incontinence. Br J Surg.

[18] Jabbar SAA, Camilleri-Brennan J (2022). An evaluation of the long-term effectiveness of Gatekeeper™ intersphincteric implants for passive faecal incontinence. Tech Coloproctol.

[19] Gassner L, Wild C, Walter M (2022). Clinical effectiveness and safety of self-expandable implantable bulking agents for faecal incontinence: a systematic review. BMC Gastroenterol.

[20] Ratto C, Buntzen S, Aigner F, Altomare DF, Heydari A, Donisi L (2016). Multicentre observational study of the Gatekeeper™ for faecal incontinence. Br J Surg.

[21] Litta F, Parello A, De Simone V, Campennì P, Orefice R, Marra AA (2020). Efficacy of Sphinkeeper™ implant in treating faecal incontinence. Br J Surg.

[22] de la Portilla F, Reyes-Díaz M, Maestre M, Jiménez-Rodríguez R, García-Cabrera A, Vázquez-Monchul J (2017). Ultrasonographic evidence of Gatekeeper™ prosthesis migration in patients treated for faecal incontinence: a case series. Int J Colorectal Dis.

[23] Trenti L, Biondo S, Noguerales F, Nomdedeu J, Coret A, Scherer R (2017). Outcomes of Gatekeeper™ prosthesis implantation for the treatment of fecal incontinence: a multicenter observational study. Tech Coloproctol.

[24] Brusciano L, Tolone S, Del Genio G, Grossi U, Schiattarella A, Piccolo FP (2020). Middle-term outcomes of gatekeeper implantation for fecal incontinence. Dis Colon Rectum.

[25] Leo CA, Leeuwenburgh M, Orlando A, Corr A, Scott SM, Murphy J (2020). Initial experience with SphinKeeper™ intersphincteric implants for faecal incontinence in the UK: a two-centre retrospective clinical audit. Colorectal Dis.

[26] La Torre M, Lisi G, Milito G, Campanelli M, Clementi I (2020). Sphinkeeper™ for faecal incontinence: a preliminary report. Colorectal Dis.

[27] Dawoud C, Bender L, Widmann KM, Harpain F, Riss S (2021). Sphinkeeper procedure for treating severe faecal incontinence—a prospective cohort study. J Clin Med.

[28] Vaizey CJ, Carapeti E, Cahill JA, Kamm MA (1999). Prospective comparison of faecal incontinence grading systems. Gut.

[29] Ware JE (2002). How to score version 2 of the sf-12 health survey (with a supplement documenting version 1).

[30] Colbran R, Gillespie C, Warwick A (2022). A prospective trial of the THD SphinKeeper^®^ for faecal incontinence. Colorectal Dis.

[31] Al-Ozaibi L, Kazim Y, Hazim W, Al-Mazroui A, Al-Badri F (2014). The Gatekeeper™ for fecal incontinence: another trial and error. Int J Surg Case Rep.

[32] Grossi U, Brusciano L, Tolone S, Del Genio G, Di Tanna GL, Gambardella C (2020). Implantable agents for fecal incontinence: an age-matched retrospective cohort analysis of gatekeeper versus sphinkeeper. Surg Innov.

[33] Dawoud C, Capek B, Bender L, Widmann KM, Riss S (2021). Re-Do Sphinkeeper™ procedure for treating recurrent faecal incontinence—a video vignette. Colorectal Dis.

